# Music Therapy and Parkinson’s Disease: A Systematic Review from 2015–2020

**DOI:** 10.3390/ijerph182111618

**Published:** 2021-11-04

**Authors:** Manuel Joaquín Machado Sotomayor, Víctor Arufe-Giráldez, Gerardo Ruíz-Rico, Rubén Navarro-Patón

**Affiliations:** 1 Faculty of Education Sciences Human and Technologies, National University of Chimborazo, Riobamba 060102, Ecuador; mmachado@unach.edu.ec; 2 Department of Specific Teaching Training and Research and Diagnosis Methods in Education, University of A Coruña, 15001 A Coruña, Spain; 3 Department of Education, Educational Sciences Faculty, University of Almería, 04120 Almería, Spain; ruizrico@ual.es; 4 Department of Applied Learning, Faculty of Teacher Training, Universidade de Santiago de Compostela, 15705 Santiago de Compostela, Spain; ruben.navarro.paton@usc.es

**Keywords:** music therapy, Parkinson’s disease, review, influence

## Abstract

Parkinson’s disease can be approached from various points of view, one of which is music therapy—a complementary therapy to a pharmacological one. This work aims to compile the scientific evidence published in the last five years (2015–2020) on the effects of music therapy in patients with Parkinson’s disease. A systematic review has been performed using the Web of Science and Scopus databases with the descriptors “music therapy” and “Parkinson’s disease”. A total of 281 eligible articles were identified, which, after applying the inclusion and exclusion criteria, were reduced to 58 papers. The results display a great diversity of evidence, confirming positive effects on various spheres. All mentioned patients with Parkinson’s disease had experienced different music therapy programs. Some studies focused on the motor component, which can be addressed through listening, body rhythm, and rhythmic auditory stimulation. Other studies confirm effects on communication, swallowing, breathing, and the emotional aspect through programs that focus on singing, either individually or in groups, in order to improve the quality of life of people with PD. It was concluded that music therapy programs can achieve improvements in various areas of patients with Parkinson’s.

## 1. Introduction

Music therapy has been defined as the use of sounds and music within an evolving patient–therapist relationship to support and develop physical, mental, and social spiritual well-being [1]. Music has cognitive, psychosocial, behavioral, and motor benefits for people with neurological disorders such as dementia or Parkinson’s disease (PD) [2,3,4,5,6]. The profound value that music brings to human health and well-being provides a framework for the development of non-pharmaceutical treatments for neurological disorders [7]. In this context, making music is a powerful way to engage a multisensory and motor network, including inducing changes and linking brain regions within this network. These multimodal effects on the emotion and reward system in the brain can be used to facilitate the therapy and rehabilitation of neurological disorders [8]. A recent study confirmed that a music-based physical therapy program improved balance and functional mobility in patients with PD [9].

This disease is a neurodegenerative disorder characterized by a movement disorder, with the relative preservation of intellectual abilities [10]. According to De Bartolo et al. [11], in PD, the timing and size of repetitive sequences of internally generated automatic movements are particularly affected. The most evident consequence of this deficit is the alteration of gait patterns, including loss of rhythm, shorter steps, slower gait, and trunk instability. The most frequent non-motor symptoms, in addition to cognitive and emotional disturbances, are autonomic, sensory, gastrointestinal dysfunctions, and sleep disorders [12].

Throughout history and up to the present, music and medicine have been closely related [13]. Thus, music therapy and other musical and rhythm-based interventions can offer a variety of symptomatic benefits for patients with PD and other movement disorders [14]. Listening to different musical genres has induced modifications of spatiotemporal parameters and trunk oscillations in the gait [11]. According to De Luca et al. [15], music-based rehabilitation for gait training can be considered an effective strategy. Rhythmic auditory cues have also been established to improve gait and motor behaviors in PD and other disorders [16].

In fact, a recent systematic review and meta-analysis working with a total of 17 studies linked to music therapy and PD with a total sample of 598 participants concluded that music therapy provides an effective treatment approach to improve motor function, balance, freezing of gait, gait speed, and mental health in patients with Parkinson’s disease [17].

In voice development, a group singing program with deep breathing training and song learning can promote memory, language, speech information processing, executive function, and respiratory muscle strength in older adults, as well as those with PD [18,19]. Choral singing has also been used in this therapeutic process to help those with PD manage some of the consequences of their condition, including social isolation and low morale [20]. Given the above, music therapy in PD has been shown to have physical, emotional, and social benefits [21]. In order to continue research on the possible positive effects of programs based on music therapy as complementary to pharmacological treatment in PD patients, a systematic review has been conducted in the main databases over the last five years (2015–2020), which classifies the findings in different categories and contributions in the health of patients. Some research work, such as that of Barnish and Barran [22], has addressed the performing arts, including music therapy and its possible positive effects on PD. This study focused mostly on music therapy but also included a systematic review of the studies in general, taking into account their apparent scarcity. However, this indeed fosters a global vision of all the scientific knowledge generated to research the relationship between music therapy and PD.

## 2. Materials and Methods

Systematic review studies may serve as up-to-date knowledge troves on a topic of interest [23]. In contrast to an original research study, a systematic review of the literature analyzes the cumulative nature of scientific knowledge about a given body of knowledge, thus constituting a tool that informs and develops relevant practices and typically invites larger discussions in further academic work [24].

### 2.1. Protocol and Registration

The parameters and recommendations outlined in the PRISMA Declaration were used to carry out this review, complying with the analysis of the 27 items indicated in the declaration [25]. The review was not recorded. Review data can be requested from the authors.

### 2.2. Eligibility Criteria and Risk of Bias

To assess the risk of bias and avoid any type of bias present in the search for manuscripts and the preparation of the results, the following inclusion criteria were used, as shown in Table 1:

To contextualize what is understood about music therapy, the definition established by the World Federation of Music Therapy serves as the focal point: “Music therapy is the use of music and/or its musical elements (sound, rhythm, melody, and harmony) by a qualified music therapist, with a client or group, in a process designed to facilitate and promote communication, relationships, learning, mobilization, expression, organization, and other relevant therapeutic objectives in order to meet physical, emotional, mental, social, and cognitive needs. Music therapy aims to develop potentials and/or restore functions of the individual so that [the individual] can achieve better intra- and/or interpersonal integration and, consequently, a better quality of life, through prevention, rehabilitation or treatment” [26].

### 2.3. Information Sources and Search Combination

For the search of scientific documents we have chosen to work with two international multidisciplinary databases: Web of Science, using its complete catalog (Science Citation Index Expanded, Social Sciences Citation Index, Arts and Humanities Citation Index, Conference Proceedings Citation Index, Conference Proceedings Citation Index—Social Science and Humanities, Book Citation Index—Science, Book Citation Index Social Sciences and Humanities, Current Chemical Reactions, Index Chemicus, and Emerging Sources Citation Index), and Scopus. In addition, the following descriptors from the UNESCO Thesaurus were determined as descriptors: Parkinson’s; music therapy.

### 2.4. Search

Database searches were carried out during the first month of the year 2021, including the articles resulting from the following combination of the descriptors [“Parkinson’s” AND “music therapy”] and the selection of search fields title; keywords; abstract or subject. The age filter used was 2015–2020. Subsequently, all the references extracted were uploaded to the bibliographic manager RefWorks of Proquest© where they were filtered to find duplicates, and the registered articles were purified.

**Figure 1:**
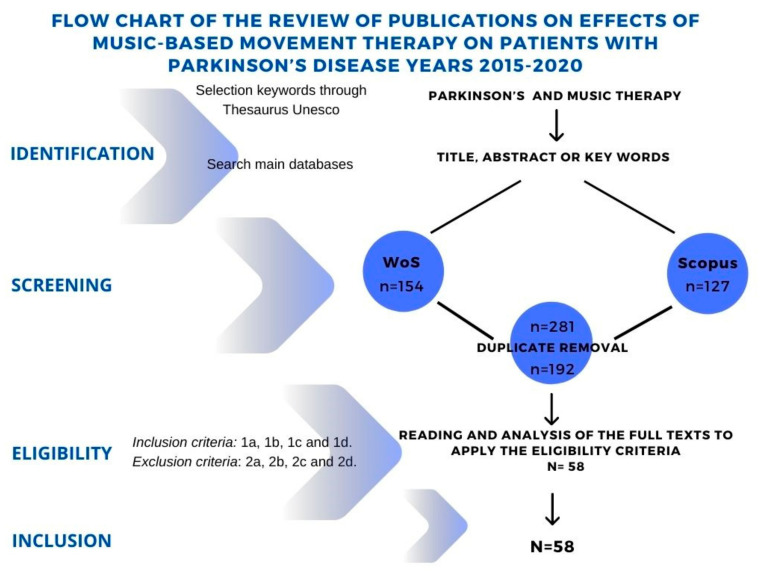
Systematic review flow chart.

The following Figure 1 shows the flow chart of the search for bibliographic references.

### 2.5. Selection of Studies

Once filtered, the references were thoroughly reviewed. Those studies that did not meet the inclusion and exclusion criteria were excluded. In turn, an Excel spreadsheet with the article data was given to an external reviewer to filter the articles by applying the inclusion and exclusion criteria, thus minimizing bias. After checking and cross-checking the data of the researchers and the external reviewer, the final data extraction was carried out.

### 2.6. Data Extraction Process and Data Listing

The detailed data and information from each selected study were entered again in an Excel spreadsheet. A total of 4 categories associated (See Table 2) with the sphere, where the effects of the music therapy program were produced in the PD patient, were established.

## 3. Results

An analysis of the 58 articles was carried out via a thorough individual reading, and research categories were identified and filtered according to the criteria of music therapy and its different treatment alternatives for Parkinson’s disease. Therefore, it has been divided into four categories (Table 3).

The following tables (Table 4, Table 5, Table 6 and Table 7) show the data related to the selected studies, highlighting the authors, study findings, sample size, mean age of the participating subjects, and the type of study. Each of the analyzed articles and the sphere or domain to which they belong are reported in four tables. Given the diversity of protocols used in the different studies, no meta-analysis or effect measurement could be performed.

## 4. Discussion

According to the results in Table 3, the articles from 2015 to 2020 have been grouped into four categories. The category regarding music therapy and movement as a treatment in people with Parkinson’s has a total of 29 articles. The other category regarding research on music therapy and communication activity in patients with Parkinson’s shows 10 publications. The category on music therapy and its influence on the emotional part of the patient with Parkinson’s has a total of 13 articles. And finally, the category on the application of music therapy in the cognitive area of the PD patient shows 6 published articles.

### 4.1. Effects of Music Therapy on the Motor Sphere

There is evidence that music therapy and other methods using music and rhythm may significantly improve a wide range of symptoms in neurological and non-neurological disorders. Devlin, Alshaikh, and Pantelyat [14], in a review of articles, highlight findings from recent studies using music and rhythm-based interventions for gait impairment, other motor and non-motor symptoms in people with PD, and other movement disorders. The limitations of current studies, as well as those of future directions that research on this topic may take, are discussed. Recent findings have demonstrated the short-term benefits of rhythmic auditory stimulation on gait parameters [27,28], including freezing in such patients, indicating that it may reduce falls, which contributes to the management and maintenance of long-term mobility [29]. The demonstration of the benefits of music in gait on “on” and “off” dopaminergic states suggests that this intervention may be a valuable addition to the current array of therapies.

An experimental study by Harrison [30] shows that singing at different tempos, either aloud or mentally, can improve gait and offers insight into how internal cueing techniques can improve motor performance for older adults and people with PD. The methodology used sixty participants over 50 years of age; 30 had PD, and 30 were age-matched healthy controls. Participants completed walking trials with internal and external cueing techniques at 90%, 100%, and 110% of their preferred cadence. The effects of different types of cues and rates were evaluated in a repeated-measures, cross-sectional study comparing gait characteristics (speed, cadence, and stride length) and variabilities (coefficients of variation of stride length, stride time, and single support time). The results of this study show that all participants modified their cadence and stride length. Thus, it is concluded that self-voiced movement improves gait characteristics while reducing gait variability for older adults and people with PD.

Rhythmic auditory stimulation (RAS) can also compensate for the loss of automatic and rhythmic movements, according to Calabrò [31]. His study suggests that the use of signaling strategies during gait training consists of remodeling sensorimotor rhythms and fronto-centroparietal/temporal connectivity. The focus is also on the restoration of internal synchronization mechanisms that generate and control motor rhythmicity, thus improving gait performance while paying special attention to individual differences in synchronization skills [32]. Regarding falls in PD, key gait parameters, such as speed and step length, were modified [33]. If this stimulation is performed in a familiar context with the support of caregivers, gait training with music may provide a feasible course to improve gait stability disorders [34].

In a translational approach, Dalla Bella [35] states that rhythmic skills are widespread in the general population. Most people can extract the regular rhythm from music and align their movements with it. To illustrate this pathway in PD, the positive effects of rhythmic auditory cues in walking are taken as a model. In Parkinson’s, a relationship is found between the success of this intervention-based music and individual differences in rhythmic skills. In addition, rhythmic auditory signals can be optimized through the use of mobile technologies in the form of dedicated apps or serious games.

In the field of auditory stimulation, different musical genres have their effect in terms of how classical music reduced the speed and trunk inclination, while the range of pelvic obliquity movements in the frontal plane increased with rock, motivational, and heavy metal songs [36]. The use of step sounds also had an improvement in spatiotemporal parameters [37]. In the same sense, auditory rhythms strengthen the stability of movement in time and space [38]. Given the above, the application of hearing for strengthening movement disorders ought to be taken more seriously [39], not only from the point of view of mobility but also from the psychological, socio-affective, and well-being point of view [40].

### 4.2. Effects of Music Therapy on the Social and Communication Sphere

Impaired communication is one of the most common symptoms in PD, significantly affecting the person’s quality of life. In a study by Tamplin et al. [41], they consider that singing shares many of the neural networks and structural mechanisms used during speech and therefore has the potential for therapeutic application to address speech disorders. They therefore set out to explore the effects of an interdisciplinary singing-based therapeutic intervention (*ParkinSong*) on voice and communication in people with PD, concluding that *ParkinSong* is an attractive intervention with the potential to increase volume and respiratory function in patients with this condition.

Hypokinetic dysarthria during the disease was analyzed with regard to communication skills as predominant factors in daily life, with the same immediately influencing decreased competence in communication, thereby increasing frustration and a loss of confidence, regardless of the degree of symptoms. Regarding the feasibility and outcome measures, they concluded that there is initial evidence to warrant further study of the protocol. On the other hand, in a narrative review, the role of music therapy in improving aphasia and other neurological disorders was described, underlying the reasons why this tool could be effective in rehabilitation settings, especially in people affected by stroke [42], in maintaining vocal skills, and in delaying the vocal deterioration that often accompanies PD [43].

There is a need to provide therapy for voice, breathing, and swallowing difficulties because current pharmacological and surgical treatments do not effectively treat these deficiencies, so the use of group singing is a good alternative [43]. Previous research has shown that singing can be a treatment option to detect voice, breathing, and swallowing problems as well as the quality of life [44]. Regarding food intake, group singing was found to be important in prolonging laryngeal elevation, protecting the airway from foreign material for longer periods during swallowing [45]. Group singing is also important for the improvement of language [19,20], in the correction and maintenance of vocal function, as well as in respiratory pressure [44]. In a study by Rojas Romero [46], an important effect on the intensity and diadochokinesia of speech was observed as well.

### 4.3. Effects of Music Therapy on the Emotional Sphere

PD is a complex diagnosis commonly associated with motor dysfunction, but it is also known to encompass cognitive, psychiatric, and mood disorders. Music has been successfully used to address motor and non-motor symptoms. Morris [47] administered two surveys to 19 people with PD and 15 people without PD to assess their subjective impressions and appraisals of music. They concluded that people with Parkinson’s may perceive less of an automatic connection between music and activity than their healthy peers. In addition, those with PD may receive more pleasure and value from music than they anticipate. Taken together, these results suggest that people with PD may require encouragement to participate, as well as to the ability to choose familiar selections to better access music-based interventions and the benefits they may offer. This may facilitate adherence to therapy, as music is engaging and enjoyable [34], improves mood, depressive syndromes [48], and thus improves the quality of life for people with PD [49,50].

Depression often accompanies the patient, which is the most common mental disorder of any person with PD, especially those with non-motor symptoms. It not only aggravates the patient’s movement and cognitive impairment but also severely affects their quality of life; therefore, attention should be paid to the antidepressant treatment of these patients with a depressive disorder based on neurological singing [51]. Antidepressant medications are indeed the most reliable procedures, but for various reasons, quite a few convalescents have not been treated with antidepressants [52]. Therein lies the importance of music therapy for PD to experience increased motivation, self-confidence, improved motor and gait control, mood regulation, improved focus, emotional expression, and support regarding the therapeutic relationship [53]. There is also the combination of music therapy and physical therapy, which, according to Bashir [54], had a greater effect on depression. This is corroborated by Stegemöller [55], who states that participants who benefit have a positive impact on their lives.

### 4.4. Effects of Music Therapy on the Cognitive Sphere

Spina [56] detected a beneficial effect of music therapy on cognition. There was an improvement at the end of the program in tests that examine frontal lobe function (cognitive flexibility, processing speed, attention, and working memory) in a pilot study conducted on Parkinson’s patients, which suggests that the music-based intervention could improve frontal function by acting as a training ground for these cognitive skills since it stimulates attention and executive functions such as planning, flexible thinking, and execution. Although, according to the authors, this effect tends to disappear after the music therapy program is stopped, so it should be continued for a longer period.

However, another study led by Dalla Bella [35] shows how patients with relatively moderate rhythmic abilities are the most likely to benefit from rhythmic auditory signals. These can be enhanced with the use of technological devices such as cell phones and tablets, among others, which can help to generate, apart from rhythm, the correction of cognitive functions of speech and language. This applies not only to people with PD but to patients such as children and adults with neurodevelopmental disorders.

In contrast, other authors have used the effects of group singing with the participation of eleven patients with a diagnosis of PD (mean age 70.6 years). According to the participants, group singing positively affects their quality of life, and there was an improvement in their physical and mental being, as well as an improvement in social connectedness and sense of self in cognitive functioning [57]. Music therapy itself, besides having direct effects on motor skills and memory, appears to be a therapy to strengthen cognitive abilities [58].

Finally, Lesiuk [59] demonstrates that piano training, based on fine finger movements, activates the cerebellum and, in comparison to other previous research on musical training, helps improve executive functions, i.e., working memory and processing speed in both healthy adults and adults with cognitive impairment, as well as in patients with PD. This is corroborated by Bugos [60], whose research aims to evaluate the impact of a novel intensive group piano training program on executive functions in PD patients. The intensive piano training consisted of basic piano technique, finger dexterity exercises, basic piano repertoire, and music theory over 10 days. Participants completed a battery of standardized cognitive measures of processing speed, cognitive control, and verbal fluency, pre-, and post-training. From the above, it can be stated that intensive piano training can serve as an effective cognitive and psychosocial intervention for people with PD.

## 5. Conclusions

This review aimed to analyze the effects of music therapy programs on patients with Parkinson’s disease. A total of 58 scientific papers were analyzed and the following conclusions were drawn:

- The positive effects of music therapy programs on different spheres of human development in Parkinson’s patients are confirmed.
- The vast majority of studies have addressed the motor component, stimulating it through music therapy programs based on listening, body rhythm, and rhythmic auditory stimulation.
- The studies that have analyzed the components of communication, swallowing, breathing, and the emotional aspect coincide with the importance of music therapy with the use of individual or group singing in order to optimize the quality of life of the patient with PD.
- There are few studies on the application of music therapy that analyze the cognitive part of the patient, and that should be treated with a procedure that helps to maintain or improve this aspect.
- It is essential to continue with scientific research to establish and perfect therapeutic processes using music therapy, with its various mechanisms, to alleviate the symptoms of people with PD.

## 6. Study Limitations

Although it has many strengths, this systematic review also has some weaknesses. One of them is related to the databases used. Two of the great world databases that bring together millions of scientific documents have been used, but it would be advisable for future revisions to also search more databases.

The descriptors used can also influence the total number of articles selected to synthesize the information. Therefore, the authors suggest using different combinations of new terms related to music therapy and Parkinson’s disease.

The concept of music therapy can also have multiple meanings. In our work, we have tried to unify the term following the definition of the World Federation of Music Therapy. But even so, some studies use different connotations of this term, which can modify the results found in the bibliographic search.

Lastly, it should be noted that the conclusions presented in this study are always contextualized to the protocol used for the writing of this systematic review study.

## Figures and Tables

**Table 1:** Inclusion and Exclusion Criteria.

Inclusion Criteria	Exclusion Criteria
1.a. Scientific papers published in the form of peer-reviewed scientific articles	2.a. Publications that do not have access to at least the abstracts
1.b. Research of any kind (experimental, review, descriptive, etc.)	2.b. Music therapy is not part of the treatment of PD patients
1.c. Research on music therapy for people with PD	2.c. Documents that are not published in the form of a peer-reviewed scientific article: for example, theses, conferences, editorials, opinion articles, etc.
1.d. Publications indexed in databases between 2015–2020 provided they are in the English language, at least in their title, abstract, and keywords.	2.d. Duplicate items

**Table 2:** Categories established for the analysis of the selected articles.

Category 1	Effects of music therapy on the motor sphere
Category 2	Effects of music therapy on the social and communication sphere
Category 3	Effects of music therapy on the emotional sphere
Category 4	Effects of music therapy on the cognitive sphere

**Table 3:** Categories of analysis of the scientific literature and number of published articles.

Category	Number of Items	Authors and Years
Effects of music therapy on the motor sphere	29	Akramova and Rakhimbaeva, (2019); Andrejeva et al. (2019); Ashoori et al. (2015); Bashir et al. (2017); Begel et al. (2017); Bella et al. (2015); Bella et al. (2018); Bellinger et al. (2016); Bienkiewicz and Craig (2016); Bukowska, (2016); Calabrò et al. (2019); Chomiak et al. (2017); De Bartolo et al. (2019); De Luca et al. (2020); Gondo et al. (2019); Harrison et al. (2017); Harrison et al. (2018); Hellman et al. (2019); Hove and Keller, (2015); Hu, (2016); Lindop and Skelly (2018); Murgia et al. (2018); Pereira et al. (2019); Thaut, (2015); Varlet et al. (2018); Veron-Delor et al. (2019); Wittwer et al. (2019); Zhang et al. (2017).
Effects of music therapy on the social and communication sphere	10	Azekawa and Lagasse, (2018); Barnish et al. (2016); Harris et al. (2016); Higgins and Richardson, (2019); Majdinasab et al. (2017); Matthews et al. (2018); Monroe et al. (2020); Stegemöller et al. (2017); Tanner et al. (2016); Tamplin et al. (2019).
Effects of music therapy on the emotional sphere	13	Bae and Mijung (2016); Berger et al. (2019); HyunJuChong, (2020); Juraev et al. (2019); Panebianco and Lotter, (2019); Pohl et al. (2020); Raglio et al. (2015); Schiavio and Altenmüller, (2015); Stegemöller et al. (2017); Trost et al. (2018); Wu et al. (2015); Young Han et al. (2018); Zhang and Li, (2019).
Effects of music therapy on the cognitive sphere	6	Boni and Cattaneo, (2016); Bugos et al. (2019); Dalla Bella, (2018); Lesiuk et al. (2018); Romane et al. (2017); Spina, (2016).

Source: own elaboration.

**Table 4:** Articles that address the effects of music therapy on motor function.

Author/Country	Year of Publication	Sample Size	Mean Age	Type of Study	Results
Akramova and Rakhimbaeva/Uzbekistan	2019	100	DNA	Intervention	Use of music improves tempo-rhythmic correction
Andrejeva et al./Lithuania	2019	M: 9 F: 9	67.5	Intervention	The application of the musical elements improved the static balance of the patients
Ashoori et al./United States	2015	N/A	N/A	Review	Use of auditory pacing improves gait disturbances
Bashir et al./Pakistan	2017	15	DNA	Intervention	Improvement in stiffness, leg agility, posture, gait, postural stability, and activity from supine to sitting position with the application of music therapy
Begel et al./France, Poland	2017	27	N/A	Review	Rhythmic musical games develop motor functions
Bella et al./France, Poland	2015	N/A	N/A	Review	Increased stride length and speed through the application of rhythmic auditory cues
Bella et al./France, Poland	2018	n/a	N/A	Review	Individualized rhythmic cues provide a safe alternative for walking
Bellinger et al./Germany	2016	EG: 26 CG:21	DNA	Intervention	The processing of time and rhythm is a Gestalt process and cortical areas involved in the processing of musical syntax can compensate for circuits
Bienkiewicz and Craig/France, United Kingdom	2016	21	N/A	Review	Music therapy is not only effective from a mobility point of view but also a psychological, socio-affective, and well-being point of view
Bukowska/Poland	2016	EG: 30 CG: 25	DNA	Intervention	Significant changes in spatiotemporal gait parameters when applying sensorimotor techniques of music therapy
Burt et al./Canada	2020	EG:15 CG:15	DNA	Intervention	Musical contingent gait training is feasible and safe in people with PD; the average adherence to training was 97%, with no falls during the training sessions
Calabrò et al./Italy, United States	2019	50	DNA	Intervention	The utility of signaling strategies during gait training consists of remodeling sensorimotor rhythms and fronto-centro-parietal/temporal connectivity
Chomiak et al./Canada	2017	11	DNA	Intervention	Wearable devices can be used to enable musically contingent SIP training to increase motor automaticity in people living with PD
De Bartolo et al./Italy	2019	60	DNA	Intervention	Post hoc analyses showed that classical music reduced trunk velocity and trunk tilt, while the range of pelvic obliquity movements in the frontal plane increased with rock, motivational, and heavy metal songs
De Luca et al./Italy	2020	EG:20 CG:20	DNA	Intervention	Musical rehabilitation based on the use of the treadmill was significant in motor functioning, concerning static and dynamic balance
Gondo et al./Japan	2019	20	DNA	Intervention	Significant improvements in stride, gait speed, cadence, acceleration, and trajectory through the use of a portable gait rhythmogram
Harrison et al./United States	2017	23	DNA	Intervention	Singing shows promise as an effective cueing technique that may be as good as or better than traditional cueing techniques for improving gait among people with PD
Harrison et al./United States	2018	90	DNA	Intervention	Internal signaling techniques such as chanting can be beneficial for walking
Hellman et al./United States	2019	EG:14 CG:14	67.79	Intervention	They challenge previous studies suggesting that variable auditory cues improve gait in PD patients
Hove and Keller/United States, Australia	2015	N/A	N/A	Review	Treatment employing groove, auditory, low-frequency, and adaptive (GABA) signals could help optimize rhythmic sensory signals to treat motor and timing deficits
Hu/Canada	2016	7 M: 5 F: 2		Intervention	Sensorimotor signaling using self-generated motor signals and music-based reinforcement learning facilitates automatic stepping behavior and sustained relief
Lindop and Skelly/United Kingdom	2018	DNA	DNA	Case Study	Music as an auditory cue may be effective in the long term in Parkinson’s, contributing to the management and maintenance of mobility, anxiety, and mood
Murgia et al./Italy	2018	38	68.2	Intervention	The use of step sounds as rhythmic auditory stimulation improves spatiotemporal parameters
Pereira et al./Brazil, United States	2019	45	N/A	Review	Music therapy interventions combined with dance are simple, non-invasive treatment options that promote walking and cognition
Thaut/United States	2015	N/A	N/A	Review	The comprehensive effects in improving multiple aspects of motor control established the first neuroscience-based clinical approach to music, which became the basis for the subsequent development of neurological music therapy
Varlet et al./Australia	2018	22	DNA	Intervention	Auditory rhythms can be used to improve the stability of movement in time and space
Veron-Delor et al./France	2019	EG:12CG:12	DNA	Intervention	Musical sonification had a significant contribution to movement disorders
Wittwer et al./Australia	2019	5	DNA	Intervention	Gait training to music may provide a feasible approach to improving gait stability disorders
Zhang et al./China	2017	11	N/A	Review	There was positive evidence to support the use of music-based movement therapy in the treatment of motor function

Note abbreviations: EG, experimental group; CG, control group; F, female; M, men; DNA, data not available; N/A, not applicable.

**Table 5:** Articles addressing the effects of music therapy on the social and communication sphere.

Author/Country	Year of Publication	Sample Size	Mean Age	Type of Study	Results
Azekawa and Lagasse/ United States	2018	5	DNA	Intervention	Group singing exercises as a supporting protocol for vocal function, voice quality, articulatory control, and speech intelligibility
Barnish et al./ United Kingdom	2016	25	N/A	Review	Singing can benefit speech in people with PD
Harris et al./ Netherlands	2016	EG:15 CG:15	DNA	Intervention	Singing familiar melodies and improvised melodic continuations could be used to facilitate expressive linguistic prosody in PD
Higgins and Richardson/ United States	2019	M:5 F:5	DNA	Intervention	Choral singing may be a viable speech treatment for some people with PD
Majdinasab et al./ Iran	2017	10	N/A	Review	Singing therapy could be used alone or in conjunction with voice therapy in PD patients to improve breathing and phonation dysfunctions
Matthews et al./ New Zealand	2018	DNA	DNA	Intervention	Voice quality, respiratory function, glottal function, and cognition showed significant improvement with the use of group singing
Monroe et al./ Australia	2020	11	N/A	Review	Positive effects of a singing group for speech and voice symptoms in individuals with PD
Stegemöller et al./ United States	2017	24	DNA	Intervention	Therapeutic singing may be an attractive early intervention strategy to address oropharyngeal dysphagia
Tanner et al./ Canada	2016	DNA	DNA	Exploratory	Group vocal strengthening activities that include singing can help maintain vocal skills and delay the vocal deterioration that often accompanies PD
Tamplin et al./ Australia	2019	75	DNA	Intervention	*ParkinSong* is an attractive intervention with the potential to increase respiratory volume and function in people with mild to moderately severe PD

Note abbreviations: EG, experimental group; CG, control group; F, female; M, men; DNA, data not available; N/A, not applicable.

**Table 6:** Articles that address the effects of music therapy on the emotional sphere.

Author/Country	Year of Publication	Sample Size	Mean Age	Type of Study	Results
Bae and Mijung/ Korea	2016	35	64.5	Intervention	Group music therapy is effective in the psychosocial well-being of PD patients living in assisted and independent living communities
Berger et al./ United States	2019	EG:19 CG:15	67	Intervention	The results of this study suggest a particular reliance on the enjoyment of musical familiarity in PD, supporting the use of client-preferred familiar music in music therapy applications.
HyunJuChong/ Korea	2020	19	DNA	Intervention	The use of group singing elicited the following responses in the participants: emotional ventilation, psychological stability, change in self-perception, increased free time, change in lifestyle, positive attitudes towards music, and active interactions among group members
Juraev et al./ Uzbekistan	2019	6	46.7	Intervention	Performing special physiotherapy exercises with music therapy not only allows patients to improve their quality of life but also influences the work capacity and disability of patients
Panebianco and Lotter/South Africa	2019	DNA	DNA	Case Study	They experienced increased motivation, self-confidence, improved motor and gait control, mood regulation, improved focus, and the opportunity for emotional expression and support within the therapeutic relationship through a music therapy intervention
Pohl et al./Sweden	2020	EG:26 CG:20	DNA	Intervention	Group music intervention increases the mood, alertness, and quality of life in PD patients
Raglio et al./Italy	2015	25	N/A	Review	Most studies support the efficacy of music therapy and other musical interventions for improving mood, depressive syndromes, and quality of life in neurological patients
Schiavio and Altenmüller/United States, Germany	2015	N/A	N/A	Review	Musical intervention for PD offers possibilities for improving the overall quality of life of patients
Stegemöller et al./ United States	2017	20	DNA	Descriptive	This study provided further insight into how a therapeutic singing program can benefit participants who stated that it was mutually beneficial, fun, and engaging; participants appreciated the camaraderie with others with PD and offered minimal constructive criticism
Trost et al./ Switzerland, France	2018	DNA	DNA	Intervention	The study showed that music elicits slightly altered emotional experiences in patients with and without STN DBS; in particular, STN DBS seems to induce less distinct emotional responses, blurring the boundaries between complex musical emotions
Wu et al./ China	2015	EG:24 CG:24	DNA	Intervention	PD patients are not impaired in emotion recognition in music in this study; the relationship between musical emotion recognition and executive function is unclear
Young Han et al./ Korea	2018	9	DNA	Intervention	Singing therapy for PD patients showed improvements in vocal function and decreased symptoms of depression
Zhang and Li/ China	2019	DNA	DNA	Intervention	The authors affirm the value of music therapy in improving depression in PD patients

Note abbreviations: EG, experimental group; CG, control group; F, female; M, men; DNA, data not available; N/A, not applicable.

**Table 7:** Articles addressing the effects of music therapy on the cognitive sphere.

Author/Country	Year of Publication	Sample Size	Mean	Type of Study	Results
Boni and Cattaneo/ Italy	2016	DNA	DNA	Intervention	Music therapy has direct effects on motor, memory, and cognitive skills
Bugos et al./ United States	2019	45	DNA	Intervention	Intensive piano training can serve as an effective cognitive and psychosocial intervention for people with PD
Dalla Bella/ Canada	2018	N/A	N/A	Review	Rhythm and movement can be extended to remediation of cognitive, speech, and language functions in other patient populations, such as children and adults with neurodevelopmental disorders
Lesiuk et al./ United States	2018	N/A	N/A	Review	This review and rationale provide support for the use of music training to improve cognitive outcomes in patients with PD
Romane et al./ Australia	2017	11	70.6	Intervention	Group singing positively affected physical, mood, cognitive functioning, social connection, “fluid” effects, and sense of self
Spina et al./ Italy	2016	25	DNA	Pilot Study	Active music therapy has a beneficial effect on frontal function and cognition, although this effect tends to disappear after the intervention is stopped, suggesting that MT should be continued for longer periods of time

Note abbreviations: EG, experimental group; CG, control group; F, female; M, men; DNA, dat a not available; N/A, not applicable.

## Data Availability

Data sharing not applicable.
